# Global Variability in the Management of Inflammatory Bowel Disease: Towards Context-Specific Strategies

**DOI:** 10.7759/cureus.96156

**Published:** 2025-11-05

**Authors:** Jeimy M Castellanos, Rachel Cooney

**Affiliations:** 1 Medicine, Universidad Autónoma del Estado de Quintana Roo, Chetumal, MEX; 2 Gastroenterology, University Hospitals Birmingham NHS Foundation Trust, Birmingham, GBR; 3 Birmingham National Institute for Health and Care Research (NIHR) Biomedical Research Centre, University of Birmingham, Birmingham, GBR

**Keywords:** advanced therapies, conventional therapies, cost-effective, inflammatory bowel disease, innovative strategies, limited-resource settings

## Abstract

Inflammatory bowel diseases (IBD), including Crohn’s disease (CD) and ulcerative colitis (UC), are chronic, relapsing conditions with increasing prevalence worldwide, thought to be driven by socioeconomic and environmental factors. Managing IBD effectively across diverse healthcare settings remains a significant challenge, as there is no universal approach. Advances such as biologic therapies, biosimilars, and small molecule agents have improved outcomes; however, disparities in access, infrastructure, and costs persist, especially in resource-limited regions. As the therapeutic landscape expands, regional treatment hierarchies are increasingly shaped by resource availability, economic constraints, and healthcare system capacity. This narrative review synthesises current evidence and explores how resource availability influences treatment decision-making. It further discusses the potential of early diagnosis, preventive strategies, and dietary interventions to enhance patient outcomes and advance cost-effective care globally. Emphasising the importance of adaptable, patient-centred management, this narrative review aims to inform healthcare providers and policymakers on tailoring IBD care to maximise disease control and healthcare efficiency across diverse resource settings.

## Introduction and background

Inflammatory bowel diseases (IBD), which include Crohn’s disease (CD) and ulcerative colitis (UC), are chronic, relapsing inflammatory disorders of the gastrointestinal tract associated with significant morbidity and long-term healthcare costs [1, 2]. CD can involve any part of the gastrointestinal tract with transmural inflammation, leading to complications such as strictures and fistulas, while UC is limited to the colon and rectum, typically causing continuous mucosal inflammation [3]. Although patients often maintain a near-normal life expectancy, the relapsing nature of these diseases places a substantial burden on individuals and healthcare systems [1,2].

Beyond the individual and healthcare burden, the global impact of IBD continues to grow. In 2019, the global prevalence of IBD was estimated at over 4.9 million cases. Western Europe had the largest number of prevalent cases, with 1.03 million, followed by East Asia with 925,047 and North America with 920,750 [2]. Initially, these diseases were considered to be primarily confined by ethnicity and geography, predominantly affecting individuals of Western European descent [4]. However, findings from the Global Burden of Disease Study 2019 show that while the incidence has plateaued in Western countries, it is rising in newly industrialised regions such as Asia, Africa, and Latin America [2]. As a result, IBD has become a global disease. This rising incidence trend mirrors the epidemiological patterns seen in Western nations over 50 years ago and has been attributed to rapid socioeconomic growth, urbanisation, and lifestyle changes, with environmental factors playing a key role in disease initiation and progression [1, 2, 4]. 

The expanding global burden of IBD has highlighted pronounced disparities in healthcare infrastructure, availability of advanced therapies, and long-term disease outcomes [2,3]. In regions with limited resources, constrained diagnostic capabilities, delayed treatment initiation, and restricted access to biologic therapies contribute not only to increased morbidity but also to irreversible bowel damage and disability, demonstrating that resource inequities in IBD care are clinical as much as they are economic [5].

As IBD has transitioned from a regional to a global disease, advances in treatment have become increasingly important to manage its expanding burden. Historically, IBD treatment relied on corticosteroids, aminosalicylates, and immunomodulators such as azathioprine and methotrexate. The advent of biologic therapies, i.e., anti-tumour necrosis factor (anti-TNF) agents, revolutionised IBD care, proving to be very effective but costly therapies. A more recent turning point in IBD treatment is the availability of relatively cheap biosimilar monoclonal antibodies alongside newer, expensive targeted therapies, e.g., anti-interleukin 23 agents. Biosimilars, which are highly similar yet more affordable alternatives to originator biologics, have since improved accessibility and reduced expenditure [3].

Nevertheless, the growing range of high-cost biologic and targeted therapies has placed additional strain on healthcare budgets. As IBD prevalence increases worldwide, healthcare systems face mounting pressure, emphasising the urgent need to evaluate treatment strategies, assess their cost-effectiveness, and ideally develop innovative preventative approaches to reduce costs and improve patient outcomes [2]. Cost-effectiveness means determining whether a treatment or intervention provides good value by comparing its cost to its benefits. Cost-effectiveness analysis can guide policymakers in resource allocation decisions. It assesses whether the health gains offered by an intervention are large enough relative to any additional costs to warrant adoption [5].

Amid these considerations of cost and value, clinicians are faced with an expanding therapeutic landscape and the practical challenge of selecting the most appropriate agent. The ever-increasing number of drugs available to treat IBD brings with it the quandary of which agent to use. This narrative review aims to explore how available resources and healthcare infrastructure can impact treatment decisions, resulting in different treatment hierarchies for different countries. With the target of treatment being disease control, the choice of agent to use may not be the same for each healthcare system. We will also explore new areas of interest such as preventative approaches, earlier diagnosis and dietary treatments.

## Review

Methods

This article was designed as a narrative review with the objective of synthesising current evidence on how differences in healthcare resources, infrastructure, and economic capacity influence treatment decisions for IBD across different regions. A narrative review design was chosen instead of a systematic review due to the broad and heterogeneous nature of the available literature, which spans clinical, economic, and policy perspectives.

Given that most existing evidence originates from high-income settings with limited data from low- and middle-income regions, a narrative approach allows for the integration of diverse study types and contextual interpretation of regional disparities. Therefore, as a narrative review, a Preferred Reporting Items for Systematic Reviews and Meta-Analyses (PRISMA) flow diagram was not generated.

To ensure transparency, literature searches were conducted using PubMed and Google Scholar databases from inception to August 2025. The search strategy included the following search terms or a combination thereof: Inflammatory Bowel Disease OR IBD AND Cost OR Economic OR Cost effective OR Pharmacoeconomics OR Health Care Models AND Therapeutics OR Treatment OR Biologics OR Management AND Global health OR Healthcare disparities OR Health services accessibility AND Microbiome OR Biomarker OR Dietary therapy OR Non-pharmacologic therapies AND Emerging OR Novel OR Innovative.

This search strategy was designed not only to capture cost- and resource-based differences in IBD care but also to encompass emerging, preventative, and non-pharmacologic approaches, as these represent growing areas of therapeutic innovation and accessibility. Relevant articles were also identified from the reference lists of key reviews. Filters were applied for English-language publications, prioritising studies from the last 10 years while preserving seminal works foundational to the topic. Duplicate studies were removed manually. Two reviewers (JMC, RC) independently screened titles and abstracts, resolving disagreements by consensus.

A total of 25 resources were included in the final synthesis, supplemented by additional materials such as guidelines, editorials, and online sources. This review aims to provide a comprehensive and contextually adaptable understanding of how healthcare systems can implement cost-effective, evidence-based, and innovative strategies, including preventative and dietary approaches, to optimise IBD management globally.

Current standard of care and economic burden

Direct vs. Indirect Costs of Care

**Figure 1:**
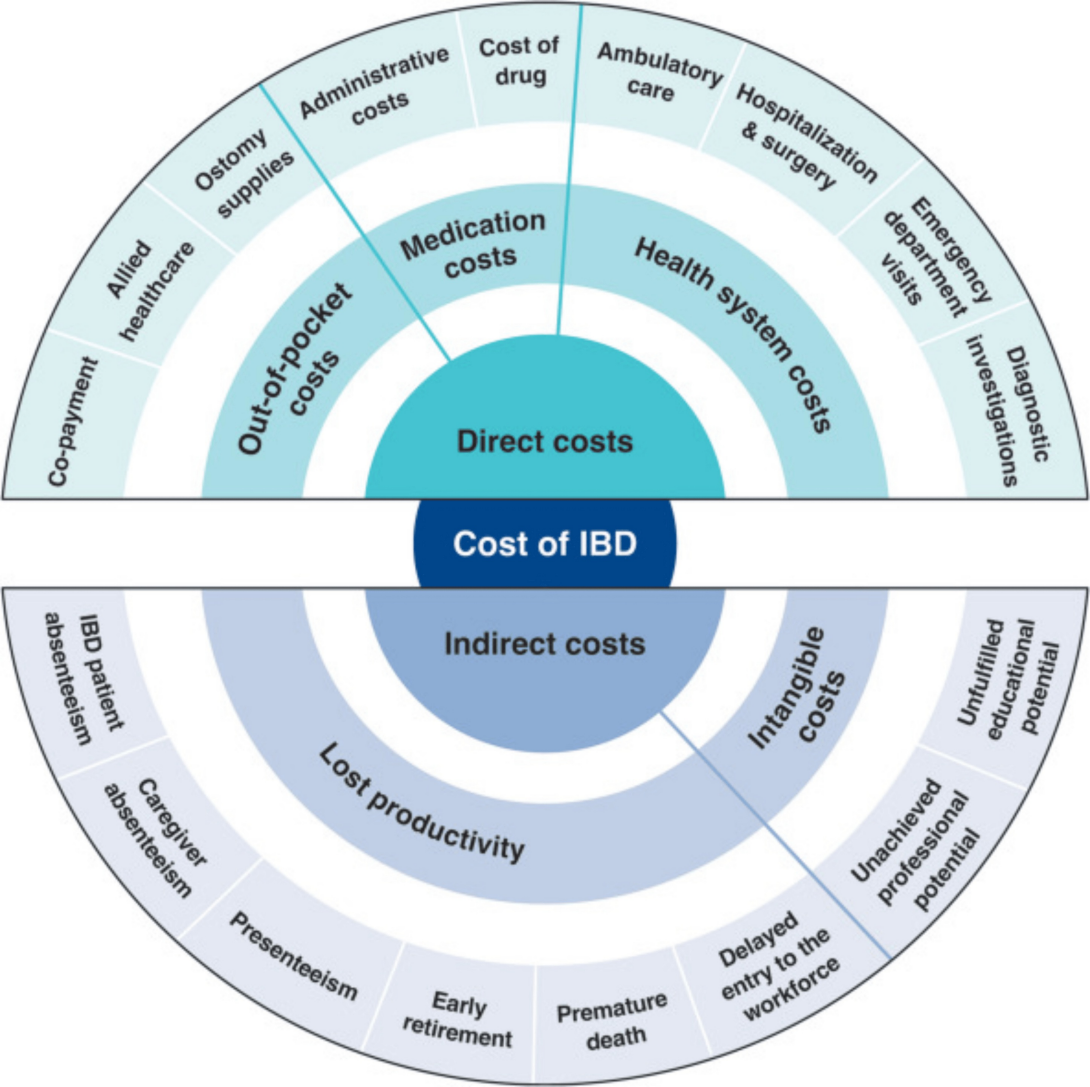
Overall economic burden of inflammatory bowel diseases (IBD), segmented into direct and indirect costs Image credit: Burisch J, Claytor J, Hernandez I, Hou JK, Kaplan GG. "The Cost of Inflammatory Bowel Disease Care: How to Make it Sustainable" @ 2025 Burisch et al., Published by Elsevier Inc. on behalf of the AGA Institute; Licensed under CC BY 4.0 [6].

Direct costs in IBD encompass the measurable medical and non-medical expenses directly associated with disease management, including medication, hospitalisations, surgeries, diagnostic investigations, ambulatory care, and out-of-pocket expenditures such as co-payments, allied healthcare, and ostomy supplies (Figure 1) [6]. Historically, hospitalisations and surgical interventions accounted for more than half of direct healthcare costs [7]. For example, a population-based cohort study across Western Europe and Israel (1993-2004) estimated an average annual cost of $2,190 per patient, with 53% attributable to hospitalisations [6]. With the introduction of biologic therapies two decades ago, cost distribution shifted markedly, as medications, particularly biologics, became the dominant driver of total healthcare expenditure [8].

Indirect costs, by contrast, capture the broader societal and economic burden of IBD, including productivity losses due to absenteeism, presenteeism, early retirement, and premature death, as well as intangible impacts such as caregiver burden and reduced educational or professional potential (Figure 1) [6]. While biologic therapies have increased direct medical costs, the advent of biosimilars and improved disease control may ultimately generate net savings by reducing indirect costs through fewer hospitalisations, decreased complications, and improved productivity. However, these indirect costs remain difficult to quantify and are often poorly defined, leading to underestimations of the true economic and societal impact of biologic and biosimilar therapies [1,6].

Regional Variations in Healthcare Expenditure

The economic burden of IBD varies widely between regions, reflecting differences in healthcare infrastructure, pharmaceutical pricing, and reimbursement systems. According to Van Linschoten et al. [8], mean annual healthcare costs for IBD, including CD and UC, range between $1,051 and $3,755 in Asia, $5,938 and $10,484 in Europe, and $8,053 and $13,212 in North America. In the UK, the National Health Service' (NHS) spends on IBD was estimated at over $1.3 billion in 2010, with an average annual cost per patient around $4,065 [9]. Multiple factors influence this difference in annual healthcare costs including pharmaceutical pricing variations and insurance models [7]. For instance, in England, the National Institute for Health and Care Excellence (NICE) evaluates the cost-effectiveness of new therapies and advises NHS England, which negotiates with pharmaceutical companies to secure favourable pricing for the public health system [10]. This centralised assessment helps regulate costs and improve access to effective treatments.

In contrast, the United States stands out for its lack of price regulation, allowing manufacturers to set high list prices. The fragmented healthcare system and extended market exclusivity periods further inflate costs, making biologic drugs several times more expensive than in other high-income countries. Although private insurers negotiate confidential rebates, patients often pay cost-sharing based on undiscounted prices, increasing their out-of-pocket burden. This lack of transparency and centralised negotiation contributes significantly to higher biologic costs in the United States compared to other nations [6].

Limitations in Cost Comparisons

It is important to note that cross-study comparisons of IBD-related costs should be interpreted with caution. The cited studies vary in design, population characteristics, time periods, and healthcare system structures, all of which influence reported costs. Additionally, currency adjustments, evolving treatment paradigms, and differences in data sources (e.g., insurance claims vs. hospital records) further complicate comparability. This heterogeneity underscores the need for standardised methodologies and up-to-date economic analyses to accurately assess the global cost burden of IBD.

Current concept of optimal IBD treatment

Modern Approach to IBD Treatment

**Figure 2:**
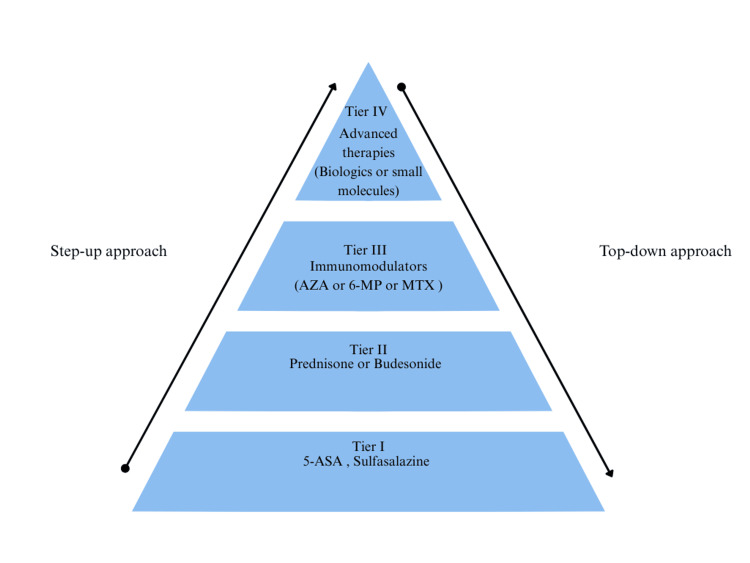
Therapeutic pyramid Step-up approach: from mild to stronger, more targeted therapies; Top-down approach: use of more potent drugs early in patient care. 5-ASA: 5-aminosalicylate; 6-MP: 6-mercaptopurine; AZA: azathioprine; MTX: methotrexate. Figure adapted from Tsui JJ, Huynh HQ. Is top-down therapy a more effective alternative to conventional step-up therapy for Crohn’s disease? © Hellenic Society of Gastroenterology. Licensed under CC BY-NC-SA 3.0 [11]. This figure has been created by the authors using Canva (Surry Hills, New South Wales, Australia).

Historically, the management of IBD followed a step-up approach, beginning with agents such as 5-aminosalicylates and corticosteroids before progressing to immunomodulators and, finally, advanced therapies such as biologics or small molecules (Figure 2) [11]. This strategy primarily focused on symptom control rather than altering disease progression. While it provided short-term relief, it often permitted ongoing inflammation, resulting in cumulative bowel damage and a higher risk of complications [6,11].

In contrast, modern guidelines advocate early, proactive intervention to achieve deep remission, both clinical and endoscopic, to prevent long-term complications such as malignancy, fibrosis, and irreversible bowel injury [7,11]. The STRIDE (Selecting Therapeutic Targets in Inflammatory Bowel Disease) and STRIDE-II consensus statements further promote a treat-to-target model, defining clear therapeutic goals such as biomarker normalisation, mucosal healing, and sustained remission, rather than focusing solely on symptom relief [12].

STRIDE provides a structured roadmap for outcome-based care, emphasising that therapy should be adjusted if these targets are not met within defined timeframes. Achieving these goals often requires earlier initiation of advanced therapies, reflecting the principles of the top-down approach (Figure 2), which aims for early and durable inflammation control to reduce surgery and improve long-term outcomes.

Evidence from Noor et al.’s multicentre open-label randomised trial on CD supports this proactive strategy, showing steroid- and surgery-free remission in 79% of patients treated with early biologic therapy compared to 15% in the conventional step-up group. The top-down group also had fewer flares, complications, and no increase in infection risk [13].

However, the top-down strategy presents challenges that limit its global applicability, including higher overall treatment costs, resource constraints, and variable access to advanced therapies. Moreover, within this strategy, selecting which advanced therapy to initiate first introduces additional considerations. Biologics and small molecules differ significantly in cost, mechanism of action, and long-term data availability, and head-to-head comparative studies remain limited. Real-world evidence on safety, treatment persistence, and outcomes across diverse populations is still evolving [6].

Therefore, while early, sustained disease control and remission remain the cornerstones of effective IBD management, therapeutic choices must be individualised, taking into account healthcare infrastructure, drug accessibility, cost-effectiveness, and the evolving evidence base rather than a rigid, universal protocol when guiding optimal selection among advanced therapies. 

Regional Disparities in the Uptake of Advanced Therapies

Despite growing evidence supporting early, disease-modifying treatment in IBD, its global implementation remains highly variable. Differences in health-insurance coverage, drug affordability, and national healthcare policy largely determine access to advanced therapies. While biologic utilisation data help illustrate these inequalities, evidence on small-molecule uptake remains even more limited, reflecting both their recent introduction and restricted availability outside high-income regions. 

In the United States, a study conducted between 2010 and 2012 reported biologic use rates of 16.8% for CD and 3.5% for UC [6]. In Central and Eastern Europe, Rencz et al. (2014) found biologic utilisation ranging from 0%-19.1% for CD and 0%-6.4% for UC [14]. Meanwhile, in the United Kingdom (UK), Selinger et al. (2024) reported biologic use at approximately 30% in CD and 15% in UC [7]. These variations reflect the influence of economic conditions, healthcare policy, and insurance reimbursement models.

In low- and middle-income countries, high out-of-pocket costs and limited insurance coverage are major barriers. The IBD Emerging Nations Consortium reports biologic use in Asia at roughly 4% for UC and 13% for CD, forcing clinicians to rely primarily on conventional immunomodulators such as thiopurines and corticosteroids, which are cheaper but less effective for long-term disease control [6,9]. A regional comparison of public health insurance systems further underscores this disparity: in Japan, where the government covers nearly all IBD-related expenses, up to 30%-40% of CD patients receive biologics, while in India, where patients typically pay out of pocket, the figure is closer to 1% [15]. 

These patterns highlight how insurance coverage, affordability, and national reimbursement frameworks are central to shaping the real-world uptake of advanced therapies, and ultimately, the feasibility of implementing a top-down strategy across different socioeconomic contexts.

Cost, Infrastructure, and Therapeutic Choice in IBD Management

In clinical practice, the selection of IBD therapies extends beyond evidence and mechanisms but is also defined by the realities of healthcare infrastructure, including the availability of infusion centres, cold-chain storage, trained personnel, and laboratory monitoring capacity. Table 1 provides a comparative overview of conventional and advanced drug classes, highlighting differences in onset of action, administration logistics, monitoring requirements, and overall cost.

**Table 1:** Comparative Checklist of IBD Drugs: Suitability for Different Healthcare Settings *(Low: <$500; Moderate: $500-5000; High: >$5000); TNFα: tumor necrosis factor-alpha; IL: interleukin; S1P: sphingosine-1-phosphate; AZA; azathioprine; 6-MP: 6-mercaptopurine; IFX: infliximab; ADA: adalimumab; JAK: janus kinase; SC: subcutaneous This table summarises the comparative characteristics of commonly used therapies for inflammatory bowel disease. Drug classes are presented as conventional therapies (aminosalicylates, corticosteroids, and immunomodulators) and advanced therapies (biologics and small molecules). Key domains include onset of therapeutic effect, logistical requirements (infusion centre, refrigeration, administration training), potential for self-administration, monitoring needs, and estimated annual cost. Cost estimates (USD) are categorised as low, moderate, or high and are intended as relative indicators rather than absolute values, as they may vary by healthcare system, availability of biosimilars, and regional pricing [16-19].

	Drug Class	Example	Onset of Action	Requires Infusion Centre	Requires Refrigeration	Requires Personal Training to Administer	Self-Administration	Monitoring	Annual Cost* (USD dollars)
Conventional therapies	Aminosalicylates	Mesalamine, sulfasalazine	Moderate	No	No	No	Yes	Frequent monitoring of full blood count, renal and liver function tests.	Low to moderate
	Corticosteroids	Prednisone, budesonide	Rapid	No	No	No	Yes	Frequent monitoring of blood pressure, blood glucose, and bone density	Low
	Immunomodulators	AZA, 6-MP	Slow (weeks-months)	No	No	No	Yes	Frequent blood counts and liver function tests.	Low
Advanced therapies: Biologics	Anti-TNFα	Infliximab, adalimumab	Rapid	IFX: yes, ADA: no	Yes	Yes (IFX: infusion training, ADA: SC injection training)	IFX-no	Monitor infections/antibodies, drug levels	Moderate (biosimilar)
	Anti-integrin agents	Vedolizumab	Moderate (weeks)	Yes	Yes	Yes (infusion training)	No	Monitor infections	High
	Anti-IL -12/ IL-23	Ustekinumab	Moderate (weeks)	No	Yes	Yes (SC injection training)	Yes, after the initial IV dose	Yes (infections)	Moderate (biosimilar)
	Anti-IL-23	Rizankizumab, mirikizumab	Moderate (weeks)	No	Yes	Yes (SC injection training)	Yes	Yes (infections)	High
Advanced therapies: Small molecules	JAK inhibitors	Tofacitinib, upadacitinib, filgotinib	Rapid (days)	No	No	No	Yes	Yes (infections, lipids, liver baseline and periodic.	High
	S1P receptor modulators	Ozanimod, etrasimod	Moderate (weeks)	No	No	No	Yes	Yes (heart rate, liver function tests, lymphocytes during first doses)	High

Treatments available and their utilisation

Traditional therapies such as thiopurines (azathioprine and mercaptopurine) and corticosteroids (like prednisolone) remain the mainstay of treatment in some regions, largely due to their low acquisition costs and ease of access [20]. Corticosteroids are inexpensive, costing roughly $106.92 annually, and effective for inducing short-term remission. However, steroids do not achieve mucosal healing [20]. Furthermore, repeated use is associated with serious adverse effects, including osteoporosis, cataracts, impaired glucose tolerance, and increased infection risk, all of which can lead to significant additional healthcare costs. Thiopurines, while useful as monotherapy maintenance in UC, are limited to use for UC only and require regular monitoring for hepatotoxicity, myelotoxicity and increased risk of pancreatitis, adding further costs and human resource burdens [9]. 

Biologics were historically associated with high costs ($14,000 to $17,000 annually [9]); however, they have become more affordable with the availability of biosimilars, reducing the price to around $6,777 for infliximab in the UK [20] and $4,470 for ustekinumab in Saudi Arabia [18]. These therapies are highly effective in inducing durable, steroid-free remission and promoting mucosal healing, which helps decrease hospitalisations and surgeries, which can translate into long-term cost savings [8]. Evidence from the NOR-SWITCH (Switching from originator infliximab to biosimilar CT-P13 compared with maintained treatment with originator infliximab) trial has demonstrated the non-inferiority in efficacy and safety of biosimilars compared to their originator biologics, further supporting their use as cost-effective alternatives [6,21]. Consequently, in the UK, infliximab biosimilars have become the preferred first-line biologic treatment, largely due to their lower acquisition costs and proven clinical equivalence [22]. However, biologics can still pose cost and logistical challenges: infusion-based agents such as infliximab and vedolizumab require specialised centres and trained personnel, whereas subcutaneous biologics like adalimumab and ustekinumab are better suited for self-administration.

The interleukin-23 (IL-23) inhibitors (such as mirikizumab, risankizumab, and guselkumab) represent a newer generation of biologics that have already been implemented in several healthcare systems. Mirikizumab, an anti-IL-23p19 monoclonal antibody, has demonstrated significant promise in achieving mucosal healing and sustained remission in both UC and CD. Similarly, guselkumab, another IL-23 inhibitor, has shown efficacy for treating moderately to severely active UC in adults who have failed anti-TNF therapy, providing a valuable second-line option. A cost analysis indicates that the expenses for guselkumab are comparable to or lower than those for mirikizumab and vedolizumab [23].

Small-molecule oral agents, including sphingosine-1-phosphate (S1P) receptor modulators (etrasimod, ozanimod) and Janus kinase (JAK) inhibitors (tofacitinib), offer unique logistical advantages. Their oral formulation eliminates the need for infusion centres and cold-chain storage, making them particularly suitable for remote or resource-limited regions. Unlike biologics, these agents are not affected by immunogenicity and can be self-administered, with simplified monitoring requirements. For example, etrasimod does not require dose titration and can be started directly at 2 mg once daily, improving adherence and minimising resource demands [19].

In addition to infrastructure, regional cost variation and healthcare financing strongly influence treatment choices. In India, generic tofacitinib is nearly ten times cheaper than the most affordable adalimumab biosimilar, making it an accessible option for UC in resource-limited settings [24]. Conversely, in high-income countries with comprehensive insurance systems, adalimumab may cost less than tofacitinib, prompting clinicians to favour it as a first-line biologic due to cost-effectiveness and familiarity. These variations underscore how economic context and health-financing structures shape therapy selection as much as clinical evidence.

Given the early onset and chronic course of IBD, early optimisation using effective advanced therapies, whether biologics or small molecules, may lead to long-term cost savings compared to less effective conventional agents [1]. The increasing use of biosimilars with proven safety profiles, such as ustekinumab, further enhances the cost-effectiveness of early, targeted treatment. As highlighted by Selinger et al. [7], timely initiation of potent therapy may allow for intermittent rather than lifelong treatment, reducing cumulative cost and exposure. The future availability of cheaper small-molecule oral agents, without the need for refrigeration, distribution networks, and the training required for biologics, and with lower monitoring requirements compared to thiopurines, will undoubtedly improve IBD care globally.

While optimising therapeutic hierarchies remains essential, attention is now shifting toward preventive strategies, early diagnosis, and proactive disease monitoring as additional cost-saving approaches.

​Prevention, early diagnosis, and monitoring

Preventive Strategies and Therapies

Preventing even a subset of IBD cases is an ambitious goal, but emerging biomarker and microbiome research suggests it may be achievable in targeted high-risk populations. Pre-disease cohorts have linked biological markers such as genetic, serological, and microbial indicators to future IBD onset. This provides an opportunity to identify individuals at moderate risk who might benefit from targeted interventions, such as microbiome modification, thus potentially delaying or preventing disease onset and reducing long-term healthcare costs [25].

Biomarker research studies, such as the GEM (Genetics, Environment, Microbial) study in Edinburgh, a prospective cohort of healthy first-degree relatives of CD patients, were groundbreaking in demonstrating that gut microbiome composition is linked to the future development of CD [26]. Initiatives like these emphasise early detection and preventive interventions that could be economically advantageous. For instance, Torres, Petralia et al.'s study identified a panel of 51 protein biomarkers that were predictive of CD up to five years before diagnosis, while no predictors could be identified for UC. This fits with the clinical observation that usually UC has a more abrupt clinical presentation and may imply a shorter preclinical period. 

However, widespread implementation of such testing remains limited by the availability, cost, and standardisation of biomarker and microbiome assays. The hope is that refining this research further will allow the development of cost-effective and scalable screening programs, allowing timely interventions to delay or modify disease onset [27].

Diet, a modifiable factor, is gaining attention as an area for preventive action, especially concerning food additives such as emulsifiers, which have become widespread since the mid-20th century [28,29]. Emulsifiers, such as carboxymethylcellulose (CMC) and polysorbate-80 (P80), are commonly used to improve food texture and extend shelf life. Recent animal studies have shown that even low concentrations of CMC and P80 can induce low-grade intestinal inflammation and, notably, promote colitis in genetically susceptible mice [28]. Similarly, a study employing the mucosal simulator of the human microbial ecosystem (M-SHIME), an in vitro model, demonstrated that both P80 and CMC act directly upon human microbiota to increase its pro-inflammatory potential [30]. In contrast, other types of emulsifiers tested showed minimal effects, highlighting the importance of identifying and limiting the most harmful food additives. Such measures could serve as a low-cost public health strategy to reduce IBD incidence among susceptible individuals [31]. For instance, recent data presented in a poster by Buckley et al. on the ADDapt trial indicate that a low-emulsifier diet (LED) is both effective and practical, while also being nutritionally safe for patients with mild to moderate active CD [32].

Biomarkers for Early Diagnosis

Biomarkers such as faecal calprotectin (FC) and serum panels have garnered significant attention due to their utility in detecting intestinal inflammation with high sensitivity and specificity. Earlier diagnosis can reduce overall costs, as effective treatments can be instituted before complications occur and reduce the need for hospitalisation. FC measurement, in particular, is widely used in clinical practice to monitor disease activity, identify relapses, and screen for suspected IBD cases. Studies have demonstrated that using FC as a diagnostic screening tool before performing invasive endoscopy can save approximately $414 per adult patient and $300 per child with suspected IBD, making it a highly resource-efficient strategy. Lowering the faecal calprotectin cutoff to 50 µg/g can reduce false negatives without substantially increasing costs [33]. Non-invasive monitoring techniques may allow for safe, non-continuous drug treatment in some individuals, again reducing drug cost [7].

Telemedicine for Easy Monitoring

Advances in telemedicine and digital health are demonstrating significant potential to reduce IBD management costs globally by improving patient monitoring and early intervention. A pilot study in the United States of America, the "IBD Medical Home" model, a multidisciplinary primary care approach that monitored high-risk patients, resulted in 47.3% fewer emergency department visits, 35.9% fewer hospitalisations, and $2500 savings per patient per year. Canadian programmes demonstrated annual savings of over $47,000, while a Dutch multicentre randomised controlled trial reported savings of around $715 per patient per year through nurse-driven telemedicine integrated with personalised treatment plans and early flare detection, maintaining quality of life [6].

In the UK, an audit evaluating a nurse-led telemedicine service for IBD at a large district general hospital over a five-month period reported net savings of $57,044.77 through avoidance of alternative care pathways such as general practitioner consultations, gastroenterology consultant appointments, accident and emergency visits, and/or hospital admissions [34]. 

While these findings highlight the value of telehealth and integrated care models in improving outcomes and reducing costs, most evidence originates from high-income countries, where infrastructure and digital literacy are more developed. Future efforts should focus on adapting and validating telemedicine models across diverse healthcare systems to ensure their feasibility and sustainability in global IBD management.

Innovative and cost-effective therapies: from diet to surgery

Dietary Interventions and Microbiome Modulation

Emerging evidence highlights the evolving role of dietary interventions, particularly exclusive enteral nutrition (EEN), in the management of IBD, especially CD. EEN, which involves providing all nutritional needs via premixed formulas administered orally or through a nasogastric tube, has been established as the first-line induction therapy for paediatric CD, given its efficacy and safety profile, and is increasingly recognised as a corticosteroid-sparing alternative in adult populations. Its efficacy in inducing clinical remission is comparable to corticosteroids, but with added benefits including superior rates of mucosal healing, reduction in disease flares, and a decreased need for surgical interventions [35]. This is particularly significant considering that up to 47% of adult CD and 16% of UC patients undergo surgery during their lifetime, a procedure associated with considerable morbidity, mortality, and long-term impact on quality of life [36]. 

Beyond its clinical benefits, EEN plays a vital role in addressing nutritional deficiencies common in CD, especially in patients with fistulising disease or prior bowel surgeries. By supporting complete nutritional intake, EEN aids in correcting deficiencies, promoting proper growth in children, and maintaining remission, while also helping to prevent long-term corticosteroid-related adverse effects such as diabetes, osteoporosis, adrenal suppression, and opportunistic infections [35].

While the precise mechanisms underlying EEN’s therapeutic effects remain incompletely understood, emerging evidence suggests that its benefits may be mediated through modulation of the gut microbiota, immune responses, and intestinal barrier function, areas that continue to be actively explored. These findings reinforce the potential of diet-based interventions as cost-effective and modifiable strategies for influencing disease progression and management [35].

Although widely adopted in paediatric CD, EEN use in adults remains limited due to issues with palatability, adherence, and long-term tolerability, as well as access to a dietician. Despite higher initial costs compared to corticosteroids, EEN may offer long-term cost savings by reducing hospital visits, decreasing the frequency of gastroenterologist consultations, and potentially delaying or avoiding costly surgical interventions. However, formal cost-effectiveness data and detailed cost analyses for EEN remain limited, particularly across different healthcare systems. The cost-effectiveness of combination strategies, such as EEN with biologic therapy, also remains insufficiently established. Prospective, real-world studies are needed to validate modelled economic benefits, quantify long-term savings, and determine the most cost-effective approaches for integrating EEN into diverse healthcare settings. Combining EEN with biologic therapies such as infliximab has shown promise in inducing and maintaining remission in selected adult cases; however, additional clinical research is required to confirm the cost-effectiveness and long-term benefits of such combination strategies [35].

Surgical Innovations: The Role of Appendectomy

While dietary interventions and pharmacotherapies remain cornerstones in the management of IBD, innovative surgical strategies are gaining attention, particularly for UC. Among these, appendectomy has emerged as a potential therapeutic strategy [37].

Recent studies, including those by Heuthorst L. et al., have highlighted the role of appendectomy in UC management. These studies assessed histological features of appendices in UC patients through clinical trials such as the ACCURE (The Effect of Appendectomy on the Clinical Course of Ulcerative Colitis) trial (NTR2883), the PASSION (Appendectomy for Therapy-Refractory Ulcerative Colitis: Pathological Improvement of Colonic Inflammation) study, the COSTA (COlonic Salvage by Therapeutic Appendectomy) study (NCT03912714), and off-label appendectomies. Findings from this research confirmed appendiceal inflammation in over 50% of UC patients, aligning with existing evidence of appendiceal involvement in UC. Significantly, it was the first to report a high rate (62.9%) of ulcerative appendicitis in patients with inactive colonic disease [37]. 

These results suggest that the appendix might play a more active role in disease progression, potentially serving as a source of ongoing inflammation rather than just a reaction to colonic activity. Because the appendix may be less responsive to topical treatments, it could contribute to the reactivation of colitis. Patients with active UC who underwent appendectomy as a therapeutic strategy showed better response rates, particularly those with ongoing appendiceal inflammation and those suffering from proctitis or left-sided UC [37].

Currently, appendectomy for UC is limited to clinical trials and is not yet a standard surgical practice. However, the insights gained present new avenues for research and could potentially lead to cost-effective treatment options. As a simple, safe, and low-cost procedure, appendectomy holds significant promise for UC treatment globally, including in both high- and low/middle-income countries. Further research to elucidate the mechanism of action of appendectomy in UC could enhance patient selection, optimise outcomes and integrate smoothly with existing medical and dietary therapies. This comprehensive approach could improve UC management, making it both effective and economically viable [37].

Limitations

As a narrative review, our approach enhances clinical readability but may be less reproducible than systematic methodologies; nevertheless, we mitigated this by reporting our timeframe, databases, keywords, and eligibility filters and by balancing seminal references with recent advances [3,11-13,17,19,20,22,25,26,27,29,31-37]. Additionally, the majority of included studies originate from high-income countries, which may limit the generalizability of our findings to low- and middle-income regions. Heterogeneity in study design, patient populations, healthcare systems, and outcome measures further complicates direct comparisons. Finally, by restricting the search to English-language publications, some relevant regional data may have been missed, and evidence regarding emerging or non-pharmacologic therapies remains preliminary.

## Conclusions

The rising prevalence of IBD worldwide underscores the urgent need for management strategies that balance clinical effectiveness with economic sustainability. While advancements such as biologics, biosimilars, and personalised monitoring tools have significantly improved outcomes, particularly in high-income countries, disparities in access, driven by healthcare infrastructure, drug pricing, and insurance models, remain a major challenge, especially in resource-limited settings. Addressing these inequities requires targeted policy measures, increased research funding, and the development of context-specific, scalable care models that are both effective and affordable. Early diagnosis, preventive strategies, and dietary interventions should be incorporated into standard care to improve disease control and reduce long-term costs.

As the primary goal is sustained disease remission, treatment choices must be tailored to what healthcare systems can feasibly deliver, rather than rigidly following guidelines designed for well-resourced environments. The most appropriate therapy is the one that patients can access, afford, and adhere to regardless of regional constraints, thus maximising disease control and quality of life. Ultimately, effective IBD management will never be entirely universal but must be adapted to different healthcare contexts, ensuring that each patient receives the best possible care within their specific setting. Fostering a global commitment to health equity, integrating comprehensive economic assessments into treatment decisions, and promoting flexible, patient-centred approaches are essential steps toward achieving sustainable, equitable, and effective management of IBD worldwide.
